# *Xenopus* embryonic epidermis as a mucociliary cellular ecosystem to assess the effect of sex hormones in a non-reproductive context

**DOI:** 10.1186/1742-9994-11-9

**Published:** 2014-02-06

**Authors:** Patricia Castillo-Briceno, Laurent Kodjabachian

**Affiliations:** 1 Aix-Marseille Université, CNRS UMR 7288, IBDM, Campus de Luminy Case 907, 13288 Marseille Cedex 9, France

**Keywords:** Cellular ecosystem, Ciliogenesis, Mucociliary epithelia, Sex steroids, *Xenopus*

## Abstract

**Background:**

How important are sexual hormones beyond their function in reproductive biology has yet to be understood. In this study, we analyzed the effects of sex steroids on the biology of the embryonic amphibian epidermis, which represents an easily amenable model of non-reproductive mucociliary epithelia (MCE). MCE are integrated systems formed by multiciliated (MC), mucus-secreting (MS) and mitochondrion-rich (MR) cell populations that are shaped by their microenvironment. Therefore, MCE could be considered as ecosystems at the cellular scale, found in a wide array of contexts from mussel gills to mammalian oviduct.

**Results:**

We showed that the natural estrogen (estradiol, E2) and androgen (testosterone, T) as well as the synthetic estrogen (ethinyl-estradiol, EE2), all induced a significant enhancement of MC cell numbers. The effect of E2, T and EE2 extended to the MS and MR cell populations, to varying degrees. They also modified the expression profile of RNA MCE markers, and induced a range of “non-typical” cellular phenotypes, with mixed identities and aberrant morphologies, as revealed by imaging analysis through biomarker confocal detection and scanning electron microscopy. Finally, these hormones also affected tadpole pigmentation, revealing an effect on the entire cellular ecosystem of the *Xenopus* embryonic skin.

**Conclusions:**

This study reveals the impact *in vivo*, at the molecular, cellular, tissue and organism levels, of sex steroids on non-reproductive mucociliary epithelium biogenesis, and validates the use of *Xenopus* as a relevant model system in this field.

## Introduction

**Figure 1:**
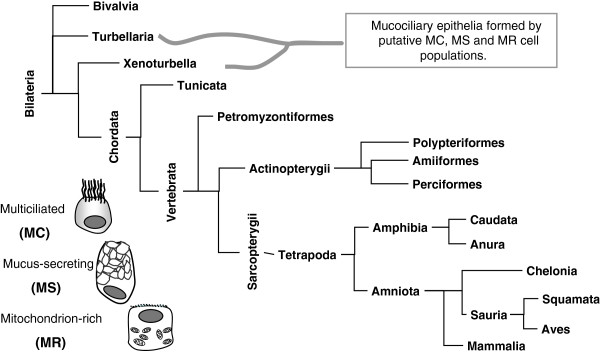
**Phylogeny of mucociliary epithelia.** The scheme includes all taxa, which contain at least one species that has been reported to have a mucociliary epithelium formed by MC, MS and MR cell populations, or by intermediate cell types, independently of the organ or tissue context.

An ecosystem is defined as a biological system composed of all the organisms found in a particular physical environment, interacting with it and with each other [1]. Animal tissues can be considered as complex cellular ecosystems composed of different cell types that can be affected by mutual interactions as well as by the elements of their microenvironment. Under this perspective, the animal skin is particularly interesting because of its direct relationship with endogenous and exogenous environments. In aquatic animals with mucosal skin, the protective role of epidermis as a barrier includes the regulation of ionic homeostasis by mechanical, molecular and cellular processes that are common to non-epidermal ion-transporting mucosa. For example, the processes of membrane Na + trafficking by mitochondrion-rich (MR) cells via phosphorylation of the Na+,K+,2Cl- co-transporter (NKCC1) and the activation of apical anion-channels in skin, gills and kidney in marine fish species, are conserved in renal and airway epithelia of amphibians and mammals [2]. Moreover, structural features of animal mucosal epithelia are also remarkably similar along metazoan phylogeny and between organs. Mucociliary epithelia (MCE) have a minimal common cellular composition with mucus-secreting (MS) and multiciliated (MC) cells, and an additional population of MR cells featured with abundant microvili, ridges and/or apical vesicles, which play a role in the ionic/gas balance. This kind of MCE cellular ecosystem has been reported in numerous biological contexts from mussel gills to tetrapod oviducts [3-16] (Figure 1, Additional file 1). Xenacoelomorpha species also display MCE features, such as the larval and adult epidermis of *Xenoturbella* (Xenoturbellida), which is composed of MS cells and MC cells with abundant mitochondria and microvilli [17], and the planarian (Platyhelminthes, Turbellaria) epidermis composed of MC-MS cells and ion-sensitive cells [18,19]. Multiple biological functions can be carried out by mucociliary epithelia, including the production of currents for food ingestion [3,5] or gamete transport [15,16], animal displacement [18], protection from exogenous noxious substances by mucus clearance [3,13,14] or mucus trapping [13], and ionic balance in response to serotonin [5] or gases [5,19]. MCE features are also generated in non-physiological contexts, such as induced ciliogenesis via androgen/anti-estrogen stimulation in mammalian female prostate (skene glands) [20], and the bronchogenic/foregut cysts processes in liver, renal pelvis and other organs [21,22].

**Figure 2:**
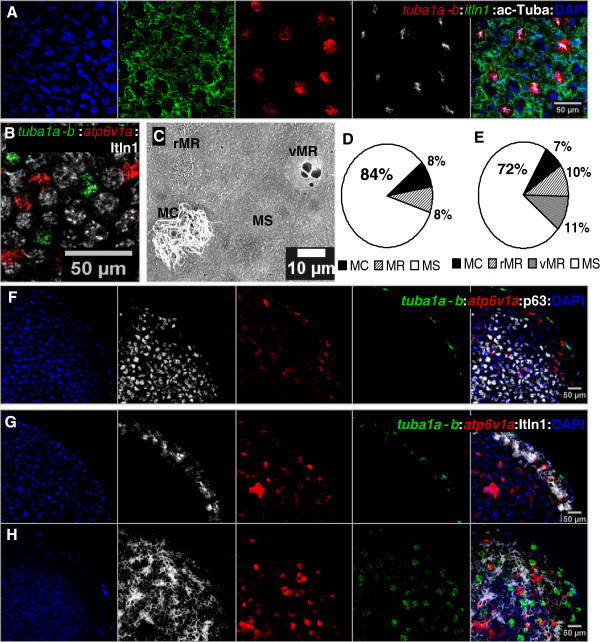
**Cellular arrangement within the mucociliary epithelium (MCE) of *Xenopus *embryonic epidermis and ectodermal explants. A,B)** Typical MCE arrangement (skin st 30) with a layer of MS cells and interspersed MC and MR cells. Cell populations are marked by fluorescent ISH and/or IHC as follows MC cells (*tuba1a-b*, ac-Tuba), MR cells (*atp6v1a*), and MS cells (*itln1*, Itln1), and imaged by confocal microscopy. **C)** SEM micrograph of skin st 40 showing the MCE cellular arrangement with MC, MR (rMR and vMR) and a predominant amount of MS cells. **D)** Abundance of MC, MR and MS cell populations as identified by biomolecular markers in skin at st 30. **E)** Abundance of MC, rMR, vMR and MS cell populations as identified by SEM in skin at st 40. **F-H)** Specified (st 15, **F)** and differentiated (st 30, **G,H)** MCE in ectodermal explants showing a similar MC, MR and MS cellular arrangement to the MCE in the skin of whole-embryos; p63 (IHC) is a marker of inner-layer basal cells in the skin. **A,B, H)** 2 μm deep stack; **F,G)** single optical sections through the explants. **H)** surface view of the explant. MC, multiciliated; rMR or vMR, ridged or vesicle mitochondrion-rich; MS, mucus-secreting.

Regarding the MCE that constitutes the *Xenopus* (Amphibia) embryonic skin, its cellular arrangement includes (i) an outer-layer of MS (also called goblet) cells intercalated by MC and MR cells, and (ii) an inner layer of basal cells that serve as a reservoir of undifferentiated cells [11,12]. The architecture of the frog MCE can be observed through *bona fide* markers of those cells types by *in situ* hybridization (ISH) and immuno-histochemistry (IHC), and by scanning electron microscopy (SEM) [11] (Figure 2A-C). Thus, it has been proposed as a model for studies aimed at comparing molecular and cellular principles of ciliated and transporting specialized epithelia [11,23]. For example, miR-449 microRNAs were reported to be key regulators of multiciliogenesis, via repression of the Delta/Notch signalling pathway, in both the *Xenopus* embryonic epidermis and the human respiratory MCE [12]. Here, we take advantage of this frog MCE model to evaluate the role of sex hormones in a non-reproductive context.

Sex steroids can drastically modify a microenvironment in reproductive organs, e.g. androgens may foster oncogenesis in prostate [24], and E2 may drive humanization of murine mammary glands [25]. Regarding the effects of sex steroids on MCE, it has been mostly studied in the oviduct tissue of tetrapods. It was reported that changes in ovarian hormones (E2, T and progesterone) transform MCE architecture and functionality linked to reproductive processes [15,16]. Moreover, exogenous E2 or T was shown to enhance ciliogenesis in oviduct MCE [26,27]. Whether these effects of sex steroids reflect a purely reproductive function or a more general impact on mucociliary cellular ecosystems remains an opened question. To address this question, we studied the effect of sex steroids on the epidermal MCE of *Xenopus* embryos. We assessed the effect of exogenous treatments with natural estrogen – estradiol (E2) – as the main active vertebrate estrogen, and testosterone (T) as the main vertebrate androgen, as well as the synthetic estrogenic compound – ethynyl-E2 (EE2), a broadly used contraceptive drug and a common pollutant of public concern because of its negative impact as an endocrine-disrupting compound (EDC). Here, it must be stressed that there is a rising number of reports about EDC pollutants, including natural sex hormones, present in surface waters, which can be bio-accumulated [28], and affect the wildlife and public health [28-31].

## Results

### Characterization of epidermal MCE architecture and differentiation during embryonic development in *Xenopus*

In the outer layer of the differentiated epidermis of *Xenopus* embryos, confocal imaging of biomolecular markers specific for MS, MC and MR cells (Additional file 2) revealed a predominance of MS cells, which form a web-like layer (Figure 2A), with intercalating MC (Figure 2A,B) and MR cells (Figure 2B). This MCE arrangement was confirmed by SEM morphological analysis, which also allowed differentiating between ridged MR (rMR) and vesicle MR (vMR) cells (Figure 2C, Additional file 2). At st 30, the MCE is composed of 7-10% MC cells, 6-10% MR cells and 66-88% MS cells (Figure 2D). At st 40, there are 6-9% MC cells, 13-28% MR cells (6-14% rMR and 7-14% vMR cells) and still a large proportion of MS cells (59-82%) (Figure 2E). Thus, the MR cell abundance showed a 2-fold increase between st 30 and st 40. When naive ectoderm was explanted at the onset of gastrulation and cultured in isolation, the architecture of the MCE was comparable to that of the embryonic epidermis at equivalent developmental stages (Additional file 3). This similarity was evidenced by internal p63 (a marker of basal cells) staining, and an outer layer of MS cells intercalated by MC and MR cells (Figure 2F-H).

**Figure 3:**
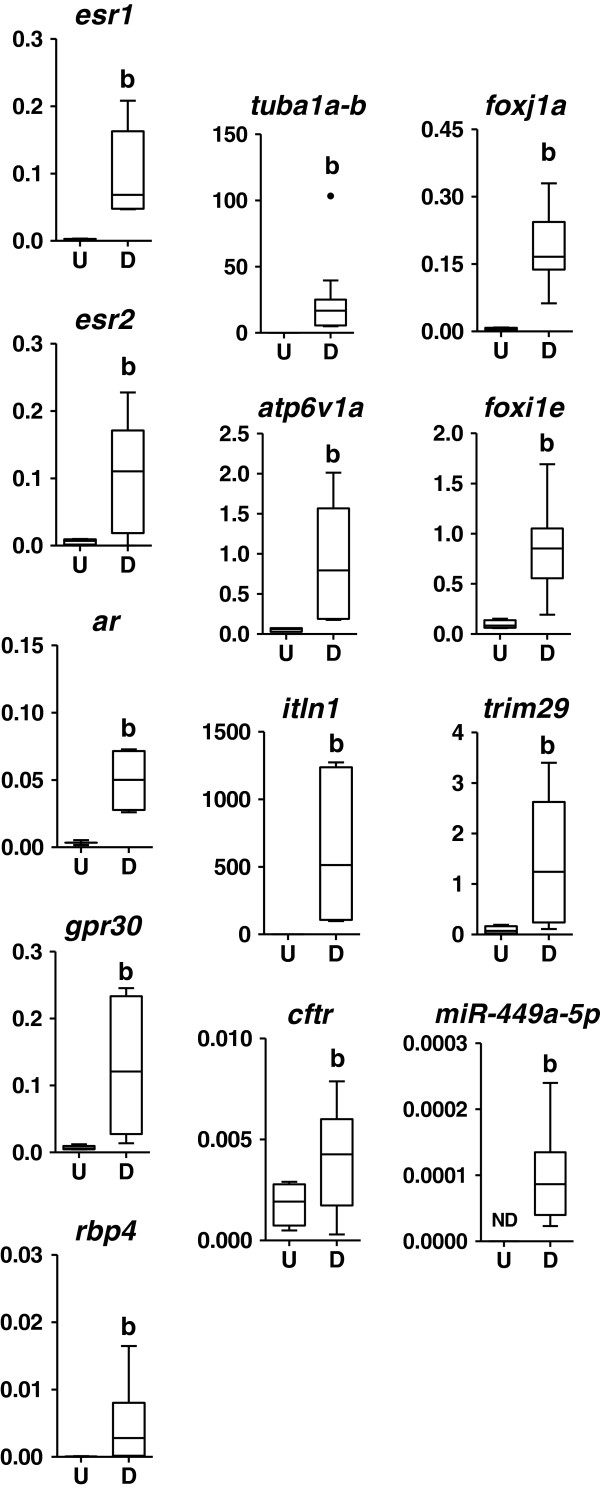
**Enrichment of marker RNAs related to sex steroid signalling and mucociliary epithelium differentiation in *Xenopus *embryonic epidermis.** Comparative analysis of undifferentiated (U, st 11) and differentiated (D, st 25–30) MCE from ectoderm explants. RT-qPCR data from pools of 20 explants each. Levels of expression are expressed relative to *odc1*, except miR-449a-5p that is relative to RNU2. Mean ± SD (boxes) and 95% CI (whiskers), outliers are represented as dots. ND = non-detected, letter “b” denotes data significantly different from (U). Two-way ANOVA, Bonferroni comparison test, p < 0.05, d.f. = 3.

We then compared temporal expression profiles of relevant marker RNAs, such as (i) androgen receptor (*ar*), estrogen receptor 1 (*esr1*), *esr2* and G-coupled protein receptor 30 (*gpr30*); (ii) the endocrine metabolism marker and plasma transporter, retinol-binding protein 4 (*rbp4*); (iii) the transcription factors related to MCE cell specification for MC cells (*foxj1*), MS cells (*trim29*) and MR cells (*foxi1e*); (iv) the multiciliogenesis regulator, the mature microRNA 449a (xla-miR-449a-5p); (v) the marker genes of differentiated MCE (Additional file 2), tubulin alpha 1a (*tuba1a-b,* MC), intelectin 1 (*itln1,* MS) and ATPase H + transporting V1 subunit A (*atp6v1a,* MR); (vi) the ABC transporter ion-channel, cystic fibrosis transmembrane conductance regulator (*cftr*), which is an important ion transporter in vertebrate mucosal epithelia. All the RNAs analyzed by RT-qPCR were detected throughout st 10 (onset of gastrulation) to st 45 (onset of gonadal development) in whole embryos (Additional file 4). Remarkably, sex steroid receptor gene expression peaked at tailbud st 20, coincidentally with the increasing expression of MCE differentiation markers (Additional file 4). In differentiated epidermis, MCE differentiation markers and the sex steroid receptors, *esr1* and *ar*, were enriched compared to whole embryo tissues at st 30 (Additional file 5). Furthermore, in explanted epidermis, all the markers, both MCE and endocrine related, were enriched in differentiated (st 25–30) vs. undifferentiated (st 11) MCE epidermis (Figure 3). This initial analysis suggested that the epidermal MCE, in early *Xenopus* embryos, is competent to respond to sex steroids.

### E2, T and EE2 effects on gross anatomical phenotype of *Xenopus* embryos

**Figure 4:**
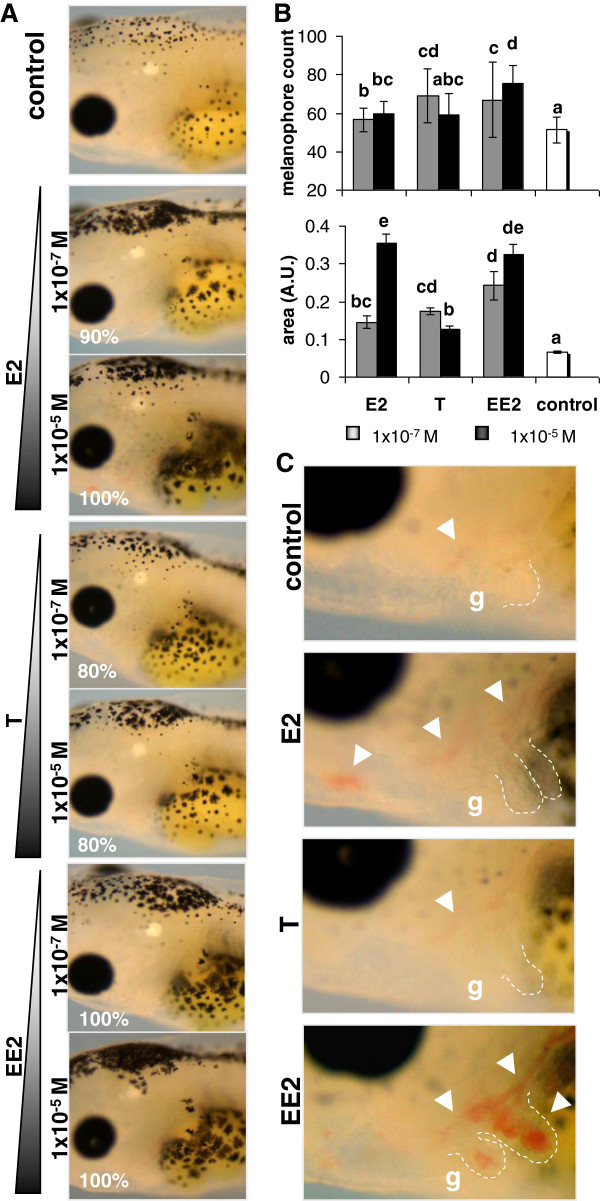
**Effects of sex steroids on the gross anatomical phenotype of *Xenopus *embryos. A)** Increase of pigmentation in the skin caused by E2, T and EE2 at indicated concentrations. Pictures of living anesthetized embryos at st 45; the percentages shown in the bottom left corner represent occurrence rates. Data are representative of 48 embryos/condition. **B)** Melanophore numbers and skin pigmented area associated to E2, T and EE2, compared to controls. Mean ± SD, “a” = control group; statistical significance is represented in a letter code: a indicates no difference with control, all other letters indicate significant difference with the control, shared letters indicate no significant differences between the corresponding data sets. One-way ANOVA, Tukey comparison test, p < 0.05, d.f. = 21. **C)** E2, T and EE2 induced overgrowth of gill branches (g, dashed lines); E2 and EE2 induced hypertrophy of blood vessels (arrowheads) in gill branches and cardiac anlage. **B)**. Data are representative of 24 embryos/condition.

Exposure to E2, T or EE2 resulted in a dose-dependent increase of skin MCE pigmentation (Figure 4A), easily discernable from tadpole st 40. There was a general increase of melanophore numbers and of pigmented area, though T exhibited a weaker effect than the estrogens, E2 and EE2 (Figure 4B).

The anatomy of gill branches, which are fully covered by MCE, was clearly affected by sex steroids. The images from light microscopy (Figure 4C) and SEM (Additional file 6) showed that E2, T or EE2 induced a hypertrophy or an accelerated maturation of gills. This effect was observable from st 40, with the most obvious alterations in E2-treated embryos. The exposure to E2 and EE2 also induced a hypertrophy of blood vessels within the cardiac anlage and gill branches (Figure 4C). The overall development of treated individuals was otherwise normal, when body size, intestinal coiling and survival rate was assessed at late tadpole st 50. We next analysed specific MCE features in response to sex hormones.

### E2, T and EE2 increase the abundance of MC cells in *Xenopus* embryonic MCE

**Figure 5:**
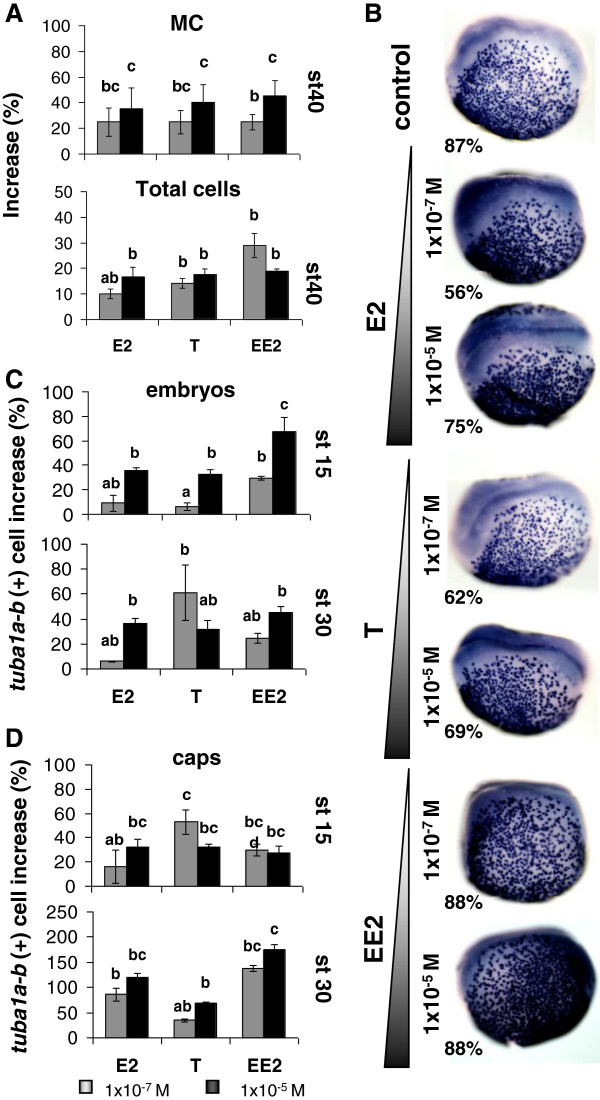
**Effect of sex steroids on the abundance of MC cells in *Xenopus *mucociliary epithelium. A)** Increase of MC and total cells by E2, T and EE2 in the skin outer layer of whole embryos st 40, SEM analysis, d.f. = 14. **B)** E2, T and EE2 increased the apparent density of MC progenitors prior to MCE differentiation (st 15), as revealed by *tuba1a-b*(+) chromogenic ISH. Images are representative of 24 embryos/condition. **C-D)** Increase of MC cells by E2, T and EE2 in whole embryos skin and ectodermal explants (caps), as measured by *tuba1a-b* (+) ISH staining of MC cells prior (st 15) and after (st 30) MCE differentiation, d.f. = 21. **A,C,D)** Mean ± SD, “a” = control group; statistical significance is represented in a letter code: a indicates no difference with control, all other letters indicate significant difference with the control, shared letters indicate no significant differences between the corresponding data sets. One-way ANOVA, Tukey comparison test, p < 0.05.

All three sex steroids induced an increase in the number of MC cells, as revealed by SEM quantitative analysis at st 40 (Figure 5A). This effect exceeded the observed increase in the total number of cells in the MCE of treated embryos, suggesting a specific impact of the sex hormones towards MC cell biogenesis. The increase of MC cell abundance induced by E2, T and EE2 was confirmed by ISH (*tuba1a-b* as MC cell marker, Figure 5B) prior to (st 15) and after (st 30) MCE differentiation (Figure 5C,D). In the embryonic skin of whole embryos, EE2 stimulated a more pronounced effect at st 15 than E2 and T; however, after longer exposure (st 30), the effect was comparable for the three steroids (Figure 5C). The enhancement of MC cells by sex steroids was also apparent in epidermal explants (caps), and even more marked in this context than in whole embryos in response to E2 and EE2 (Figure 5D).

### Sex steroids differentially affect the MCE cellular ecosystem

**Figure 6:**
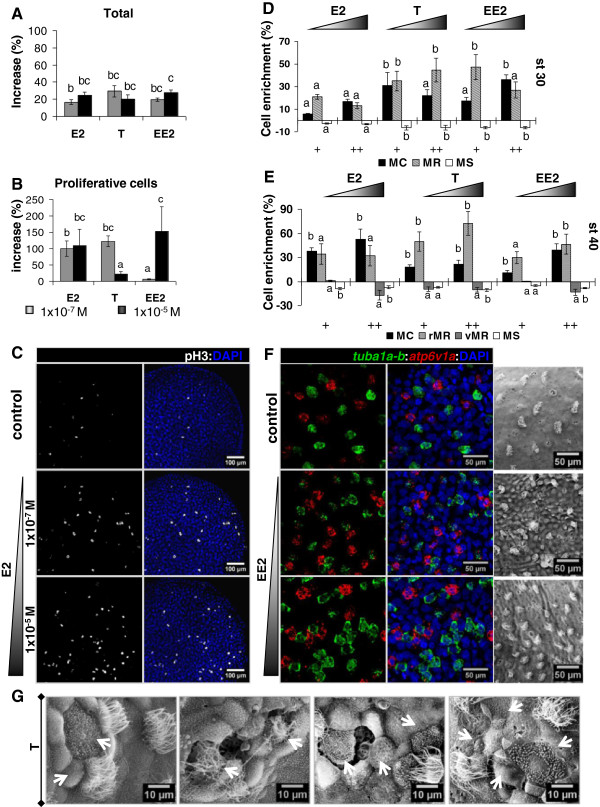
**Effects of sex steroids on the MCE cellular ecosystem. A,B)** Effects of estradiol (E2), testosterone (T) and ethynyl-E2 (EE2) on the total cell abundance in MCE, based on DAPI nuclei staining **(A)**, and on the total amount of proliferative cells (pH3 positive/field) **(B)**. Analyses by confocal microscopy in embryos at st 30, d.f. = 21. **C)** Proliferative cells in E2 treated MCE from ectoderm explants. IHC for pH3 and DAPI. Confocal microscopy, 2 μm stack. Data representative of 18 samples/condition. **D,E)** Changes in cell population enrichment mediated by sex steroids in *Xenopus* epidermal MCE. Data based on the ratio of cell counting of each population over the total number of cells at st 30 (DAPI) **(D)** or st 40 (SEM micrographs) **(E)**. Data are representative of 48 (st 30, Confocal imaging of molecular makers) or 24 (st 40) embryos by sex steroid treatment. +/++ concentrations = 1x10^-7^ M / 1x10^-5^ M. MC = multiciliated, vMR or rMR = vesicle or ridged mitochondrion-rich, MS = mucus-secreting cells. **A,B,D,E)** Baseline established for control group = 0. Mean ± SD, “a” = control group; Letter code: a indicates no difference with control, all other letters indicate significant difference with the control, shared letters indicate no significant differences between the corresponding conditions. One-way ANOVA, Tukey comparison test, p < 0.05. **F)** Disruption of cell population arrangement caused by EE2 in embryos at st 30. We observed an increase of MC (*tuba1a-b*, green) and MR (*atp6v1a*, red) cells, and the presence of adjacent MC cells. Double FISH and DAPI. Confocal microscopy, 2 μm stack, 24 embryos/condition. Right panels: Increase of the number of MC cells and disrupted epithelial architecture caused by EE2 SEM, st 40, 16 embryos/condition. **G)** Perturbed epidermal MCE arrangement and morphology provoked by T. SEM, st 40, 16 embryos/condition.

Sex steroids induced an increase of the total number of cells in the MCE, as measured by SEM at st 40 (Figure 5A, Additional file 7), and by DAPI nuclei staining at st 30 (Figure 6A), suggesting a potential impact on cell proliferation. In the interest of reproducibility, we analysed the skin over the trunk somites, where the response to sex steroids was less variable than on the abdomen, tail, head and fin areas. Using phospho-Histone H3 as a proliferation marker, it was shown that E2 consistently increased the amount of proliferative cells at both low and high concentrations (Figure 6B,C), whereas T and EE2 showed a dose-specific increasing effect on cell proliferation (Figure 6B). We next analysed the abundance of MC, MR and MS cells. E2 induced a dose-dependent increase of all three cell types at st 30 (Additional file 8) and st 40 (Additional file 7). Low and high doses of T increased the abundance of MC and MR, and to a lesser extent MS cell populations at st 30 (Additional file 8) and st 40 (Additional file 7). EE2 caused an increase of the amount of MC and MR cells at st 30 (Additional file 8), and of the amount of MC, rMR, and MS cells at st 40 (Additional file 7). vMR cell population abundance was affected only by low EE2 concentration (Additional file 7). To evaluate specific cell type enrichment in response to sex steroids, the proportion of each population was calculated as ratio to the total number of cells counted in each condition (Figures 6D,E). At late tadpole st 40, all three hormones caused a significant enrichment of MC cells (Figure 6E). E2 had no significant effects on the other cell types, except a slight decrease of MS cell percentage at st 40. Interestingly, both low and high T concentrations induced a very consistent enrichment of MR cells at st 30 and rMR cells at st 40 (Figures 6D,E). EE2 also increased MR cell percentage, though depending on the concentrations used. T and EE2 triggered a comparable decrease of MS cell percentage (Figures 6D,E). The percentage of vMR cells was not affected in any case.

Sex steroids also perturbed cellular arrangement within the MCE. Notably, E2 and EE2 caused the appearance of adjacent MC cells, which never occurs in normal conditions (Figure 6F, Additional file 9B). SEM analysis also revealed a general disrupted epithelial morphology, which was most pronounced in EE2 (Figure 6F) and T (Figure 6G) treated embryos (see also Additional file 9).

### Sex steroids alter the transcriptional expression of genes enriched in differentiated MCE and induce “non-typical” cellular phenotypes in the epidermis of Xenopus embryos

**Figure 7:**
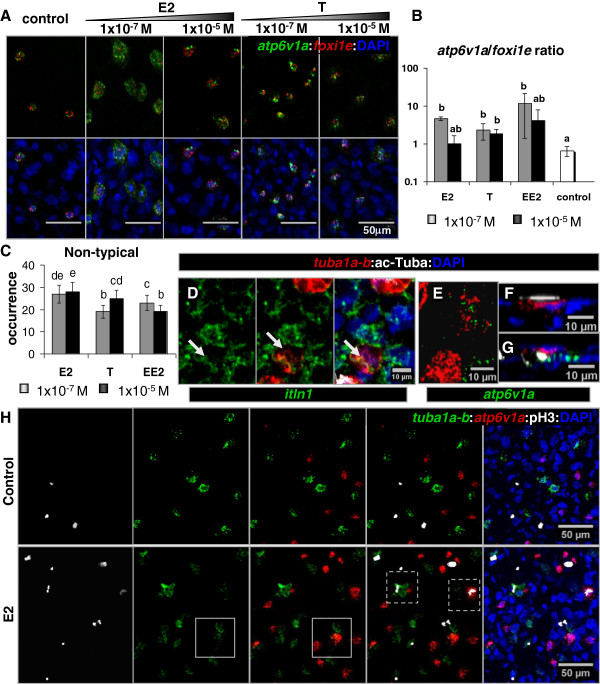
**Sex steroids induced “non-typical” phenotypes in epidermal MCE of *Xenopus *embryos. A)** Enhanced *atp6v1a* signal in MR cells treated with E2 or T. Staining: Double FISH for *atp6v1a* (green) and *foxi1e* (red), and DAPI, 2 μm stack. **B)** Quantification of *atp6v1a* enrichment based on the ratio of *atp6v1a* to *foxi1e* signal areas in single cells, d.f. = 69. **C)** Accumulative occurrence of “non-typical” phenotypes by condition. “Non-typical” phenotypes were not detected in controls. **D)** “non-typical” double *itln1*: *tuba1a-b* cell without cilia (arrow) in an EE2-treated embryo. Staining: Double FISH for *itln1* (green) and *tuba1a-b* (red), IHC for ac-Tuba, and DAPI, 2 μm stack. **E)** “non-typical” double *atp6v1a*: *tuba1a-b* cell in a T-treated embryo, 2 μm stack. **F,G)** Orthogonal view of a normal MC cell **(B)** and “non-typical” double *atp6v1a*: *tuba1a-b* cell with internalized cilia in a T-treated embryo. **B-D)** Staining: Double FISH for *atp6v1a* (green) and *tuba1a-b* (red), IHC for cilia marker acetylated-tuba (ac-Tuba), and DAPI. **H)** Induction of proliferative MR and MC (dashed-line squares), and double *atp6v1a*: *tuba1a-b* cells (square). Staining: Double FISH for *tuba1a-b* (green) and *atp6v1a* (red), IHC for proliferation marker phospo-histone3 (pH3), and DAPI, 2 μm stack. **A,D-H)** Data from confocal microscopy imaging analysis. **C-H)** “non-typical” data corresponds to a screening of 64 embryos by condition. **B,C)** Mean ± SD, “a” = control group; statistical significance is represented in a letter code: a indicates no difference with control, all other letters indicate significant difference with the control, shared letters indicate no significant differences between the corresponding data sets. One-way ANOVA, Tukey comparison test, p < 0.05. MC = multiciliated, vMR or rMR = vesicle or ridged mitochondrion-rich, MS = mucus-secreting cells.

The effect of E2 on mRNA expression levels was generally weaker than those of T and EE2, as measured by RT-qPCR (Additional file 10). The three steroids showed a tendency to down-regulate the expression of endocrine signalling related markers. In contrast, the transcriptional expression of RNAs related to MCE differentiation was distinctly affected by each hormone. The effect of E2 was restricted to MR cell markers, with a slight inhibition of *foxi1e* and an enhancement of *atp6v1a*. However, in controls as well as in treated embryos, double ISH analysis revealed that those MR cell markers were always found co-expressed (Figure 7A). Interestingly, the *atp6v1a*/*foxi1e* ratio showed a marked increase in cells treated with E2 (Figure 7B). The same tendency was apparent in embryos treated with T and EE2, albeit with more variability in the latter case (Figure 7A,B). The other RNAs (*tuba1a-b*, *itln1*, *foxj1a*, *trim29*, miR-449a-5p and *cftr*) were not significantly modified by E2 treatments. For T and EE2, the effects were variable depending on the markers. Treatment with T tended to up-regulate *tuba1a-b*, miR-449a-5p (MC) and *atp6v1a* (MR) markers and down-regulate *iltn1* and *trim29* (MS) markers. Treatment with EE2 inhibited *foxi1e* and *trim29* expression, whereas it enhanced miR-449a-5p and had no effect on *tuba1a-b*, *atp6v1a*, *itln1* and *foxj1a*.

All sex steroids also caused a remarkable induction of “non-typical” cellular phenotypes. The occurrence of such aberrant phenotypes was higher in E2 than in T or EE2 treated embryos (Figure 7C). However, the impact on the overall tissue appeared to be stronger with EE2 and T than with E2 (Additional file 9, Figure 6F,G). There was a wide array of “non-typical” phenotypes based on molecular markers and morphological features (Additional file 11). Among them, we found intermediate MC-MS (Figure 7D) and MC-MR (Figure 7E) cells, abnormal MC-like cells with apparently internalized cilia or non-ciliated (Figure 7G), proliferative MC (pMC) and pMR (Figure 7H), and abnormal MS-like cells or grouped MR cells (Figure 6G).

Taken together, the collected evidence indicates that sex steroids cause profound defects in MCE architecture, cellular composition and molecular profiles.

## Discussion

Here, we conducted a case study to assess the effects *in vivo* of sex steroids on MCE in a non-reproductive biological context. Taking advantage of the accessible MCE of tadpole epidermis, we have shown that sex steroids have a profound impact on normal MCE development. Notably, this study is the first to report that E2, T and EE2 increase the abundance of MC cell population in a non-reproductive MCE (Figure 5). Conversely, E2, T and EE2 induced strong transformations in the tadpole MCE resembling those caused by endocrine disruption in genital mucosal epithelia [15,16,27]. Furthermore, E2 and T effects detected at molecular, cellular and tissue levels in *Xenopus* embryos are comparable to EE2 effects, documenting the risk of E2 and T as potential EDCs for amphibians and other vertebrates.

In the physiological context, it has been shown that endogenous concentrations of E2 and androgen are naturally high in *Xenopus laevis* embryos before gonadal sex differentiation [32]. Moreover, we showed that sex steroid receptor genes have a prominent expression before the onset of sexual differentiation (Additional files 4 and 5) and that they are upregulated in differentiated MCE (Figure 3). Thus, we propose that sex steroid signalling could have a role during early development of *Xenopus*, independently of the reproductive system.

In sex steroid stimulated embryos, the overall developmental features were preserved. However, prominent and specific changes in skin pigmentation were observed (Figure 4). Consistent with these observations, previous studies showed that natural estrogens may affect melanin containing cells by increasing their abundance [33,34], enhancing the tyrosinase activity [34,35] and increasing the melanosome transfer via CFTR and ESRs signalling regulation [35]. CFTR expression was also shown to be regulated by estrogens in mucosal epithelia [36,37], affecting pH, fluid volume and transport in the tissue [38]; in tadpole epidermis, we showed that *cftr* was upregulated by the synthetic EDC, EE2. Moreover, androgens have been linked to the regulation of mucosal pigmentation induced by anti-malarial drugs [39] and of tyrosinase activity in melanocytes via cell membrane signalling [40]. Our study thus suggests that skin pigmentation may be used as an obvious external parameter to assess endocrine stimulation. Moreover, the measurement of skin pigmentation together with the morphological analysis of gills and blood vessels appeared to be a good non-invasive test for a rapid diagnostic of endocrine disruption in amphibians.

The enhancement of MC cell abundance in *Xenopus* tadpole MCE by E2 (Figure 5A-D) indicates that E2 induces this typical response not only in the reproductive MCE of amniotic vertebrates [16,26,27]. This observation is also consistent with the loss of cilia induced by estrogen deficiency in the laryngeal mucosa of rats [41]. We found that the amount of MC cells is also increased by T and EE2 (Figure 5A-D), It has been shown that a T enhances ciliogenesis and MC cell amounts in female gerbils prostate mucosa [20], and that a natural increase of T and E2 is linked to more MC cells in oviduct epithelia of tetrapods [16]. Regarding EE2 effect on MC cells, there are some reports suggesting a possible association to ciliogenesis perturbation in endometrial tissues [42,43] and in respiratory mucosa after long exposure to high doses [44]. Summarizing, the MC cell increase triggered by E2 or T in MCE of reproductive organs in amniotic vertebrates appears to be conserved in a non-reproductive amphibian tissue; effect that is also triggered by the synthetic steroid, EE2.

Our study revealed that E2, T and EE2 may affect the MCE cellular ecosystems in other aspects that the abundance of MC cells. E2 has a remarkable effect on MCE proliferative activity (Figure 6B,C), even in specified MC and MR cells (Figure 7H), which never occurs in normal conditions. Thus, it is likely that E2 perturbs MCE development through its effect upon proliferation and the resulting increase of cell abundance. E2-mediated increase of proliferative activity has also been shown on melanocytes [34], oviduct epithelia [26], respiratory mucosal endothelial cells [45] and mammary mucosal cells [25]. Conversely, T displayed more cell-type dependent effects. Strikingly, T caused a robust enrichment of MR cells (Figure 6D,E), in common with EE2 but not E2. Moreover, T and EE2 similarly disrupted the overall arrangement of the MCE cellular ecosystem in the tadpole skin (Figure 6F,G, Additional file 9A,F-I). Comparable to that overall disruption, in gerbil female prostate mucosa, T was shown to induce cell proliferation, tissue differentiation, secretory activity, dysplasia [46], enhanced ciliogenesis and MC cell differentiation [20].

Sex steroids consistently enriched the cellular expression of *atp6v1a* (Figure 7A,B), which encodes a mediator of intracellular acidification prior to endocytosis [47], suggesting a potential impact on cellular trafficking. In eukaryotic ciliated cells, endocytosis is one of the main mechanisms for internalization of ciliary components during recycling and removal of membrane material [48]. We suggest that in response to sex steroids, inappropriate endocytosis of cilia occurred in *tuba1a-*b(+) MC cells that ectopically expressed *atp6v1a* (Figure 7G). This view is consistent with multiple studies which showed enhancement of endocytic activity in response to endocrine stimulation via ATPases regulation and through multifunctional receptors [49-52].

The three steroids induced a general transcriptional inhibition of sex steroid signalling receptors, which may be part of a mechanism to cope with excess of hormones and/or their derivatives, as suggested in other models [31,53]. Consistent with this view, in tadpole st 20–38, AR and ESR lowest expression levels occurred at the time of E2 and androgen highest peaks [32]. Regarding *rbp4*, our results do not support its use as a marker to distinguish estrogenic and androgenic endocrine disruption in *Xenopus laevis*[29]. However, based on its expression, *rbp4* may be involved in MCE differentiation, in agreement with its role in fish skin patterning [54] and the role of retinoid signalling during ciliogenesis and regeneration of respiratory epithelia [55].

A spectacular effect of sex steroids in our study concerned the induction of new cellular phenotypes revealed by marker gene and SEM analyses (Figures 6F,G and 7; Additional file 9). Similar phenotypes and mixed cell identities were reported before as part of reproductive endocrine related process in oviduct epithelia [15,16], in human skene gland mucosa [56], and in stickleback fish nephritic tissues [57]. Altogether, suggesting a comparable effect among sex steroids on MCE cellular ecosystems at the cellular level. This may involve modifications of the extracellular environment [25,58,59] and/or increased cellular plasticity through inhibition of differentiation factors. In the frog skin, we note that sex steroids repressed the transcription factor *foxi1e*, which has a pivotal role during ectoderm differentiation, cell adhesion and migration [60]. We conclude that the appearance of “non-typical” cells is a useful parameter to assess the impact of EDCs on the frog MCE.

**Figure 8:**
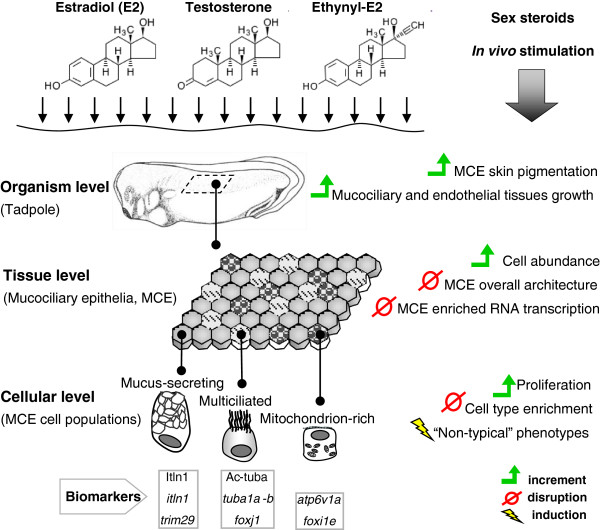
**Overview of sex steroids impact on the mucociliary epithelium of *Xenopus *** **embryos.**

Integrating our results, it can be argued that sex hormones trigger dramatic but specific changes at the organism, cellular and molecular levels in *Xenopus* embryos (Figure 8). Differences between the responses driven by E2, T and EE2 could be due to multiple and distinct mechanisms, such as the regulation of specific sex steroid receptors, or the production of various metabolites resulting from sex steroids processing [31,53]. However, it is probable that the common responses, such as enhanced pigmentation, overgrown gills, increased cellular proliferation and induction of “non-typical” phenotypes could be mediated by a multifunctional receptor with similar affinity for all three sex hormones. In this sense, membrane G-protein coupled receptors (GPCRs), such as GPR30, which was similarly inhibited by E2, T and EE2, could be good candidates. GPR30 has been shown to be signalling in response to estrogens and androgens in multiple contexts [61-64], although other GPCRs can mediate rapid and non-genomic responses to steroids in vertebrates [61,62].

In conclusion, we propose that the epidermis of *Xenopus* embryos can be used not only as a standard model of MCE cellular ecosystem, but also as a promising system for pathological and ecotoxicological assessments, and for comparative studies with other epithelial or mucosal tissue models.

## Materials and methods

### Ethics

All the experiments were performed following the Directive 2010/63/EU of the European parliament and of the council of 22 September 2010 on the protection of animals used for scientific purposes. All animal experiments were approved by the "Direction départementale de la Protection des Populations, Pôle Alimentation, Santé Animale, Environnement, des Bouches du Rhône" (agreement number E 13-055-21).

### Embryos and ectodermal explants

*Xenopus laevis* embryos were obtained from lab-bred adults (Nasco) by *in vitro* fertilization, de-jellied and cultured in modified Barth’s solution (MBS) as previously described [12], with slight modifications to maintain constant culture conditions i.e. 18°C and 0.5× MBS. Developmental stages (st) were defined based on the Nieuwkoop and Faber *X. laevis* identification keys [65], though the equivalence in hours post-fertilization differed because of the culture temperature (Additional file 3). Embryos st 8–9 were dissected to obtain explants of the animal pole central area; explants were kept 30 min. in 1× MBS in glass-petri dishes to facilitate healing and closure, and subsequently cultured in the same conditions as sibling embryos.

### Conditions for sex steroid treatments

The sex steroid hormones – E2 (17β-estradiol, Sigma), T (17β-Hydroxy-3-oxo-4-androstene, Sigma) and EE2 (17α-ethynyl-estradiol, Sigma) – were prepared following the same procedure. The hormones were directly resuspended in 0.5× MBS in an ultrasonic bath (Fisher Scientific 15046) to improve dissolution [31] at a final concentration of 1 × 10^-4^ M. Embryos and epidermal caps from st 10 were exposed to E2, T or EE2 at 1 × 10^-7^ M and 1 × 10^-5^ M by bath, kept at 18°C and collected at the timing equivalent for st 15, 30 or 40 (Additional file 3) as needed. Controls were incubated in 0.5× MBS and collected at the same time as treated embryos and ectodermal explants.

### Samples collection and fixation

Prior to fixation, embryos at all the sampled stages were placed in glass vials and anesthetized with tricaine (also called MS-222 or ethyl 3-aminobenzoate methanesulfonate, Sigma) at 0.3 mg/ml in 0.5× MBS for at least 10 min. Explants were treated accordingly to keep identical conditions.

Embryos (st 15 and 30) for biomolecular marker staining were fixed with formaldehyde 4% v/v final concentration for 2 h, then placed in 100% ethanol for 1 h; both steps at room temperature (RT) with continuous soft agitation; embryos were then placed in fresh ethanol 100% at -20°C for at least 24 h and until needed. Embryos (st 40) for SEM were fixed in 3% glutaraldehyde in 0.1 M phosphatase buffer pH 7.4 (19 ml monosodium phosphate 0.2 M and 81 ml disodium phosphate 0.2 M) made with filtered (0.22 μm) bi-distilled water, during 4 h with vigorous agitation, then washed with phosphatase buffer and filtered bi-distilled water, to be successively dehydrated in ethanol at 25, 50 and 70% for 30 minutes each; then, embryos were stored in fresh ethanol 70% at 4°C for 1–2 days before further processing.

### Bright field microscopy observations

Embryos were regularly observed in bright field 1-10× magnification for the assessment of gross morphological phenotype. No obvious differences between sex steroid-treated and control embryos were detected regarding speed of development or deformities, except for changes in pigmentation, gill branches and blood vessels. To assess these changes, embryos at st 40 and 45 were anesthetized (as described above) for static imaging of embryos alive (Digital Sight DS-L2, Nikon), the resulting pictures were further analyzed.

### SEM processing and imaging

Embryos in 70% ethanol were further dehydrated with vigorous agitation in ethanol once at 90% and twice at 100% for 30 minutes each; they were subsequently subjected to CO_2_ critical point drying (CPD030, Balzers) at 31°C and 73 atm. Finally, samples were sputter-coated with gold (vacuum 1×10^-12^ Torr, beam energy 3–4 keV) for immediate SEM digital imaging (S440, Leica) of the skin epidermis, to allow cell populations and tissue architecture analysis.

### Fluorescent and chromogenic *in situ* hybridization (ISH)

Whole-mount chromogenic ISH was performed as previously referenced for *X. laevis* epidermal markers [11,12,66], with some modifications. All products and reagents were purchased from Roche or Sigma, unless stated otherwise. RNA probes were synthesized and labelled with digoxigenin (DIG) or fluorescein (FLUO) from plasmids containing the appropriate hybridizing sequences for the intended target genes *tuba1a-b* (gift from Christopher Kintner, Salk Institute for Biological Studies, U.S.), *itln1* (gift from John Wallingford, University of Texas at Austin, U.S.), *atp6v1a* (gift from Nancy Papalopulu, University of Manchester, U.K.) and *foxi1e*; accession number of sequences are the same as for RT-qPCR primers (Additional file 12); sense probes were also synthesized and served as negative controls. Briefly, samples stored in ethanol at -20°C were progressively rehydrated in 75%, 50% and 25% ethanol in PBT (0.1% Tween 20 in 1× PBS), and then in PBT; they were then treated with 0.1 M triethanolamine (TEA) pH8 for 5 min. and 0.5% acetic anhydride in 0.1 M TEA for 10 minutes, and washed in PBT; they were then treated with proteinase K (PK) at 2 μg/ml final concentration for 8 min., washed with PBT and placed in bleaching solution (600 μl RNase free water, 325 μl H_2_0_2_, 50 μl formamide and 25 μl 20X SSC) under bright light, washed and re-fixed in formaldehyde 4% for 20 min. After that, samples were successively placed in HM hybridization mix (1% w/v Roche blocking agent, 25% 20X SSC, 50% Formamide, 0.01% heparin, 0.1% Torula RNA, 0.1% Tween 20, 0.1% CHAPS, 5 mM EDTA pH8, in bi-distilled water) 50% in PBS and HM 100%; samples were subsequently incubated with the respective probes in HM 100% at 60°C for 18 h. On day 2, samples were successively washed in HM 50% (in 2× SSC and CHAPS 0.1%) at 37°C, in 2× SSC and CHAPS 0.1% at 37°C, in 0.2× SSC and CHAPS 0.1% at 60°C, MABX (0.1% TritonX-100, maleic acid 0.1 M and NaCl 0.15 M) 50% in 0.2× SSC and CHAPS 0.1% at RT and, finally, in MABX. Embryos were then placed in blocking buffer (2% Roche blocking reagent, 10% fetal bovine serum and 5% DMSO in MABX) during 1.5 h and then incubated with anti-DIG or anti-FLUO labelling antibodies from sheep at 4°C for 18 h and washed with MABX.

For chromogenic ISH (CISH), TEA treatment was omitted. CISH was carried-out with alkaline phosphatase (AP) conjugated antibodies at 1:5000 in blocking buffer, and detected with 0.5× BM-purple substrate in water. Fluorescent ISH (FISH) was performed with horse-radish peroxidase (POD) conjugated antibodies at 1:500 in blocking buffer and detected with TSA Plus fluorescein or Cy3 kits (PerkinElmer) for FLUO or DIG tagged probes, respectively. Samples were incubated with the TSA substrate for 1 h and then washed with MABX; then, the reaction was stopped with 2% H_2_O_2_ in PBS for 0.5 h and washed in MABX. From TSA substrate addition until imaging, samples were kept protected from the light. For double ISH, the RNA probes were added at the same time, but the antibody and substrate reactions were performed sequentially. At the end, the stained samples were fixed with 4% formaldehyde in PBT for 0.5 h and, then, washed in PBT and processed for imaging or for additional staining.

### Fluorescent immuno-histochemistry (IHC)

Whole-mount IHC was performed as previously referenced in *X. laevis*[11,12,66], with some modifications. After ISH staining, samples were placed in blocking buffer and, then, incubated with the respective primary antibody for marker proteins at the corresponding dilutions in blocking buffer for 1 h at RT and then for 18 h at 4°C. We used mouse monoclonal anti-acetylated-Tuba clone 6-11B-1 (T7451, Sigma) for cilia at 1:1000, mouse monoclonal anti-p63 (ab111449, Abcam) for basal cells at 1:100, mouse monoclonal anti-Itln1 (5G7 antibody, gift from Saguro Nagata, Japan Women’s University, Japan) for MS cells at 1:500, and rabbit polyclonal anti-phospho-Histone H3 (06–570, Upstate Biotechnology) for mitotic cells at 1:1000. Then, samples were washed 7 times in MABX for 1 h at RT and for 18 h at 4°C. After that, samples were placed in blocking buffer for 1 h at RT and, then, incubated for 3 h with the appropriate anti-rabbit or anti-mouse secondary antibody made in donkey, goat or chick and conjugated to Alexa-fluor 488, 555 or 647 (Invitrogen), as needed and with the concentrations indicated by the manufacturer. Finally, samples were washed and processed for imaging or for additional staining.

### DAPI staining for nuclei

After ISH and IHC, and just before mounting, samples were placed in DAPI (Calbiochem) at 1 μg/ml final concentration in PBS for 15 min. at 4°C and then, washed two times with PBT for 1 h at 4°C.

### Fluorescent and confocal imaging

Whole embryos were analyzed by bright field, fluorescent (MZ FLIII, ebq 100 mercury lamp, Leica) and macroconfocal (AZ-C2+, Nikon) microscopy for a general screening of the epidermal architecture and verification of staining quality. Then, epidermal tissue over somites was explanted and mounted with Fluoromount G (Fluoprobes) and let to dry at 4°C before confocal imaging (LSM780, Zeiss). The images were acquired as 8 bit/channel and 1024×1024 pixel resolution, and processed in Image J for max intensity Z-projection and/or merge of channels. The enhancement of *atp6v1a* was calculated as the ratio of *atp6v1a* to *foxi1e* signals by single cell, the measurements of area of *atp6v1a* and *foxi1e* were obtained separately using Image J. The setting for acquisition and image processing were maintained between samples of the same experiment.

### Pigmentation measurements

Images from bright field microscopy observations were analyzed to assess changes in pigmentation that were quantified as 1) the number of melanophores on the abdominal area, by manual count and “find maxima” tool of Image J software [67], and 2) the total pigmented area, eyes and cement gland excluded, by using the Image J threshold tool. Both parameters were measured in embryos between st 40 and 45 when the pigmentation was sufficiently defined, so as to be detected by the software, although qualitative changes in the pigmentation were readily observable from st 38.

### Analysis of RNAs expression by real-time PCR (RT-qPCR)

Tissue samples of whole embryos, ectodermal explants and embryonic skin were frozen in liquid nitrogen and stored at -80°C for further processing. Total RNA was extracted using miRCURY RNA Isolation Kit - Tissue (Exiqon) and treated with DNase I (New England Biolabs) following manufacturer instructions. First strand cDNA synthesis was performed with SuperScript II RNase H– Reverse Transcriptase (Invitrogen) with oligo-dT_12-18_ primer from 1 μg of total RNA; for non-coding RNAs, synthesis was performed with miRCURY LNA Universal RT microRNA PCR, Polyadenylation and cDNA synthesis kit (Exiqon) from 200 ng of total RNA, all following manufacturer instructions.

RT-qPCR was performed with a CFX96 instrument (Bio-Rad) using SYBR GreenER qPCR SuperMix (Invitrogen) to analyze coding transcripts and miRCURY LNA SYBR Green master mix Universal RT (Exiqon) for non-coding RNAs. Transcripts analyzed were the endocrine related markers *ar*, *esr1*, *esr2*, *gpr30* (also called G-coupled protein estrogen receptor, *gper*) and *rbp4*, and MCE related marker RNAs *tuba1a-b*, *itln1* (also called *Xenopus* embryonic epidermal lectin, *xeel*), *atp6v1a*, *cftr*, *foxj1*, *trim29, foxi1e* and xla-miR-449a-5p.

SYBR Green reaction mixtures for coding transcripts were incubated for 10 min at 95°C, followed by 40 amplification cycles of 15 s at 95°C and 1 min at 60°C, and a final melting curve analysis verification (65 – 90°C, increment at 0.5°C/s). For non-coding RNAs analysis, the same settings were applied but with a ramp rate of 1.6°C/s during amplification cycling. The reference RNAs used were ornithine decarboxylase 1 mRNA (*odc1*, average Cq records 22 ± 1.1) for gene expression and small nuclear RNA U2 (RNU2, average Cq records 19 ± 0.7) for xla-miR-449a-5p expression. *X. laevis* specific primers were designed using the Primer-BLAST tool [68] from the sequence databases publicly available or selected from previous studies. Accession numbers, references and sequences are shown in Additional file 12. In all cases, each PCR was performed in triplicate and from at least two independent experiments. The quality of each couple of primers was tested, in all the cases the PCR product produced one single band of the expected size in Sight DNA Stain (Euromedex) 1% agarose gel, had ≥99% identity with the intended sequence, produced a single melting curve peak and had a linear curve efficiency with r > 0.95.

### Estimation of MCE cell population features

Images from confocal microscopy of embryos at st 30 stained by double FISH and IHC of molecular markers, were used to estimate the cell population abundance based on the three main cell types (MC, MR and MS cells) that can be differentiated with this approach (Additional file 2). In all cases, *tuba1a-b* was used to estimate the number of MC cells, while other gene and protein markers were combined to obtain a set of overlapping staining to estimate the amount of the other cell types and specific features (examples in Figure 2). All these data were integrated to analyze the cell population abundance (amount of cells by field), and to assess the contribution of each cell type based on its enrichment with respect to the total number of cells (amount of DAPI stained nuclei). Furthermore, ac-Tuba and pH3 were used as markers to characterize additional features on the cellular activity. In addition, multiple marker staining was also used to identify “non-typical” cell phenotypes that were quantified as accumulated occurrence. This is a similar approach to the one previously described for assessing heterogeneous cellular responses to perturbations [69]. Complementarily, the images from SEM at st 40 were analyzed to assess cellular composition based on the typical structural features of each cell type (Additional file 2), to calculate the enrichment of each cell type, and for a qualitative assessment of the general architecture of the tissue.

### Statistical analyses

Expression profiles in whole embryos were modelled from 4 data sets of independent batches of embryos with at least 20 embryos by stage (time-point) with overlapped sampling time-points from st 9–50, each time-point counting at least with 2 data sets; then, the spline-curve points ± 95% CI were calculated and plotted. Differences between time-points were analyzed by a two-way ANOVA and Bonferroni comparison test (p < 0.05). The Spearman correlation coefficient (r) and the respective p-value were calculated for each RNA marker vs. hours post hatching to estimate the changes in the expression levels associated to age (only significant for *foxi1e* (r < −0.6), miR-449a-5p (r > 0.8), *cftr* (r > 0.3), *atp6v1a* (r > 0.5) and *rbp4* (r > 0.7) profiles) and those that are more specifically related to MCE differentiation mechanisms.

For comparative analyses of RNA expression profiles from RT-qPCR data, the levels of expression were normalized and, then, analyzed by two-way ANOVA and Bonferroni comparison tests (p < 0.05). The effects of sex steroids were represented as fold-change relative to controls. Comparative analyses for pigmentation and population dynamics were performed using one-way ANOVA and Tukey’s comparison tests (p < 0.05); the effects of sex steroids were represented as percentage-change relative to controls. In all cases, differences between groups were indicated by letters in the figures or tables. Different letters denote groups that are significantly different, e.g. data group “b” is different from data group “c”, whereas a dataset labelled as “bc” is not significantly different from data group “b” or from data group “c”. The letter “a” was used to denote data that are not significantly different from the reference or control group in each experiment. Data were analyzed using GraphPad Prism v.5 or R i386 2.15.2 [70].

## Abbreviations

MCE: Mucociliary epithelia; MC: Multiciliated cells; MR: Mitochondrion-rich cells; MS: Mucus-secreting cells.

## Competing interests

The authors declare that they have no competing interests.

## Authors’ contributions

PC-B conceived the study, carried out the acquisition, analysis and interpretation of data. PC-B and LK have co-coordinated the study and drafted the manuscript. Both authors read and approved the final manuscript.

## Authors’ information

P.C-B.’s expertise is based on aquatic animal biomedicine with emphasis on its assessment at cellular and molecular levels, and using *in silico*, *in vitro* and *in vivo* approaches. P.C-B.’s scientific interests are focused on the study of potential regulators of mucosal epithelia biogenesis that are related to wound healing, immunity and endocrine control system.

L.K. has developed expertise in the role of signalling pathways during vertebrate embryonic development, using primarily *Xenopus laevis* as model system. L.K. has a keen interest in understanding ciliated epithelium biology from molecular, cellular and evolutionary point of views.

## Supplementary Material

Additional file 1 Detail of organs/tissue context in which a mucociliary epithelium formed by MC, MR and MS cells has been reported along metazoans. Click here for file

Additional file 2 **Criteria for cell type identification in the mucociliary epithelia of *Xenopus laevis *** **embryonic skin.** Click here for file

Additional file 3 **Time equivalence for Nieuwkoop and Faber developmental stages of *Xenopus laevis *** **embryos cultured at 18°C in MBS 0.5X.** Click here for file

Additional file 4 **Expression profile of marker RNAs relevant to sex steroid signalling and mucociliary epithelium (MCE) differentiation during early developmental stages of *Xenopus laevis.***
Click here for file

Additional file 5 Comparative expression profile of RNAs related to sex steroid signalling and mucociliary epithelium (MCE) differentiation in whole embryos, isolated tadpole skin or ectodermal explants. Click here for file

Additional file 6 **Effects of estradiol (E2), testosterone (T) and ethynyl-E2 (EE2) on gill branches anatomy in *Xenopus *** **embryos.** Click here for file

Additional file 7 **Effects of estradiol (E2), testosterone (T) and ethynyl-E2 (EE2) on the abundance of each cell population of the MCE in *Xenopus laevis *** **embryonic skin at st 40 (SEM analysis).** Click here for file

Additional file 8 **Estradiol (E2), testosterone (T) and ethynyl-E2 (EE2) affect the cellular composition of the *Xenopus *** **embryonic skin.** Click here for file

Additional file 9 **Effects of sex steroids on the overall arrangement of epidermal MCE in *Xenopus *** **embryos.** Click here for file

Additional file 10 Effects of sex steroids on MCE marker gene expression. Click here for file

Additional file 11 **Classification of “non-typical” cellular phenotypes caused by sex steroids in the epidermal mucociliary epithelium of *Xenopus.***
Click here for file

Additional file 12 **List of *Xenopus laevis *** **specific primers used for RT-qPCR analysis.** Click here for file
