# Supplementary material for: Xenopus embryonic epidermis as a mucociliary cellular ecosystem to assess the effect of sex hormones in a non-reproductive context
Source: Front Zool. 2014 Feb 6;11:9. doi: 10.1186/1742-9994-11-9 (PMC4015847; doi:10.1186/1742-9994-11-9)
Supplement: Additional file 1 — Detail of organs/tissue context in which a mucociliary epithelium formed by MC, MR and MS cells has been reported along metazoans. [file 1742-9994-11-9-S1.pdf]

Additional file 1. Detail of organs/tissue context in which a mucociliary epithelium formed by MC, MR and MS cells has been reported along metazoans.

Organ / Tissue context	Taxonomic groups
gills [3]	Mussels (Mollusca, Bivalvia)
endostyle [4]	Ascidians (Chordata, Tunicata)
pharyngeal cavity and gill seam [5]	Sea lampreys (Hyperoartia, Petromyzontiformes)
nephritic ducts [6, 7]	Bowfins (Actinopterygii, Teleost, Amiiformes) and seabreams (Teleost, Perciformes)
lung [8]	Bichirs (Actinopterygii, Cladistia, Polypteriformes)
pelvic kidney [9]	Salamanders (Tetrapoda, Amphibia, Caudata)
palate[10], embryonic skin [11, 12]	Frogs (Amphibia, Anura)
broncho-tracheal epithelia [12–14]	Birds, lizards, turtles and mammalian species (Tetrapoda, Amniota)
oviduct [15, 16]	Birds, lizards, turtles and mammalian species (Tetrapoda, Amniota)

MC = multiciliated, MR = mitochondrion-rich, MS = mucus secreting
