# Supplementary material for: Xenopus embryonic epidermis as a mucociliary cellular ecosystem to assess the effect of sex hormones in a non-reproductive context
Source: Front Zool. 2014 Feb 6;11:9. doi: 10.1186/1742-9994-11-9 (PMC4015847; doi:10.1186/1742-9994-11-9)
Supplement: Additional file 2 — Criteria for cell type identification in the mucociliary epithelia of Xenopus laevis embryonic skin. [file 1742-9994-11-9-S2.pdf]

Additional file 2. Criteria for cell type identification in the mucociliary epithelia of *Xenopus laevis* embryonic skin

Cell type	Morphology (SEM)	Molecular markers (*ISH*, IHC)
MC	Cells crowned by numerous cilia	*tuba1a-b* (+), ac-Tuba (+)
MR	Cells showing ridged surface (rMR) or apical vesicles (vMR)	*atp6v1a* (+); *tuba1a-b* (-); ac-Tuba (-); *itln1* (-); Itln1 (-)
MS	Cells, normally polygonal, with no evident cilia, ridges or apical vesicles	*itln1*(+); Itln1 (+); *atp6v1a* (-); *tuba1a-b* (-); ac-Tuba (-)
Total	Total count of cells in the outer epidermal layer	Total count of nuclei (DAPI) within 2 µm deep from the external surface

SEM = scanning electron microscopy, *ISH* = *in situ* hybridization for marker genes, IHC = immuno-histochemistry for marker proteins, MC = multiciliated, MR = mitochondrion-rich, MS = mucus secreting
