# Supplementary material for: Xenopus embryonic epidermis as a mucociliary cellular ecosystem to assess the effect of sex hormones in a non-reproductive context
Source: Front Zool. 2014 Feb 6;11:9. doi: 10.1186/1742-9994-11-9 (PMC4015847; doi:10.1186/1742-9994-11-9)
Supplement: Additional file 3 — Time equivalence for Nieuwkoop and Faber developmental stages of Xenopus laevis embryos cultured at 18°C in MBS 0.5X. [file 1742-9994-11-9-S3.pdf]

**Additional file 3. Time equivalence for Nieuwkoop and Faber developmental stage in *Xenopus laevis* embryos cultured at 18ºC in MBS 0.5X**

| **Stages** | **Timing** (hours post-fertilization) |
|---|---|
| 8 | 8-10 |
| 9 | 10-12 |
| 10 | 12-16 |
| 12 | 18-22 |
| 15 | 24-28 |
| 20 | 32-34 |
| 25 | 40-41 |
| 30 | 53-54 |
| 38 | 84-86 |
| 40 | 110-115 |
| 45 | 240-245 |
| 48 | 370-375 |
| 50 | 570-575 |
