# Supplementary figures and images for: Xenopus embryonic epidermis as a mucociliary cellular ecosystem to assess the effect of sex hormones in a non-reproductive context
Source: Front Zool. 2014 Feb 6;11:9. doi: 10.1186/1742-9994-11-9 (PMC4015847; doi:10.1186/1742-9994-11-9)

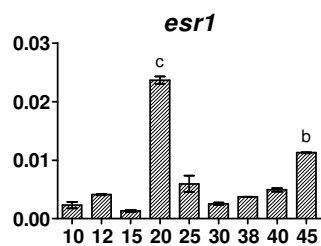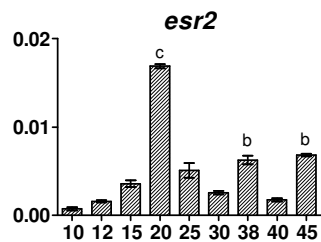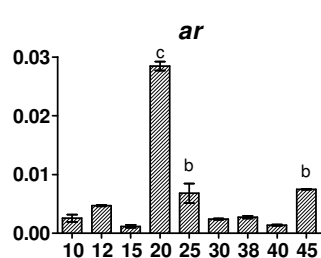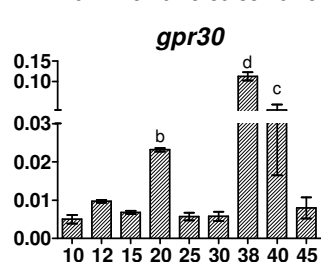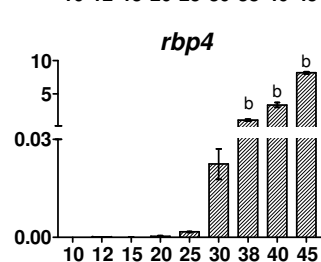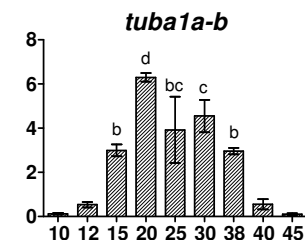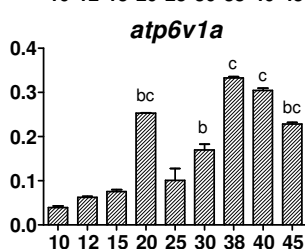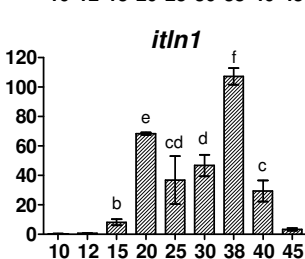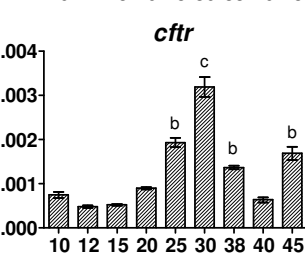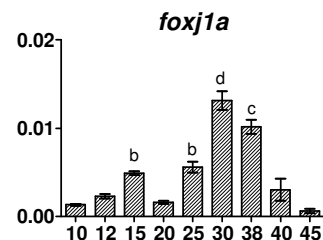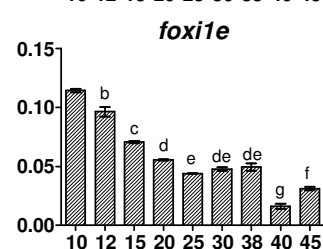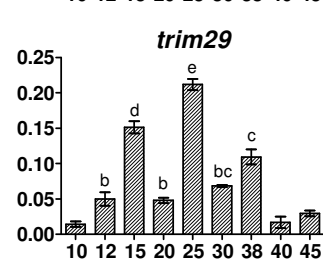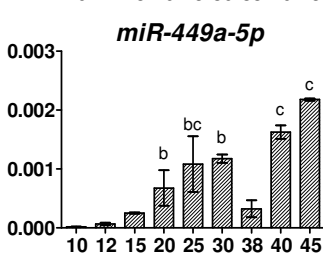

Supplement: Additional file 4 — Expression profile of marker RNAs relevant to sex steroid signalling and mucociliary epithelium (MCE) differentiation during early developmental stages of Xenopus laevis. [file 1742-9994-11-9-S4.pdf]

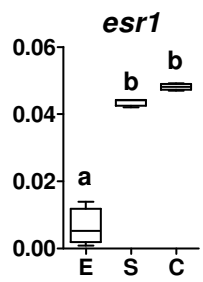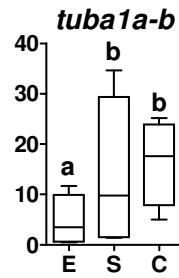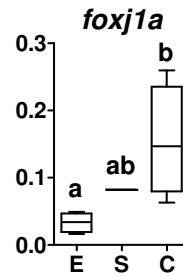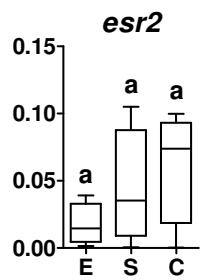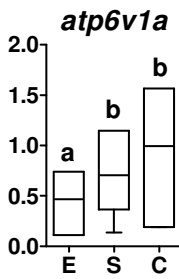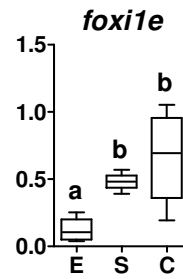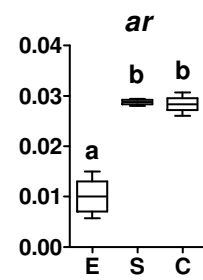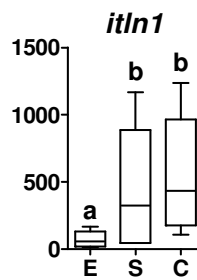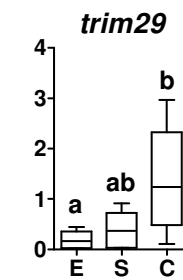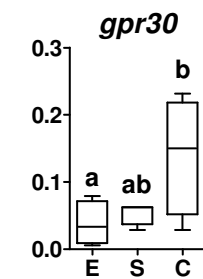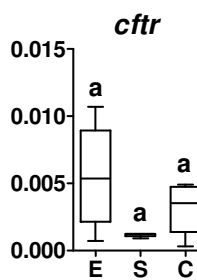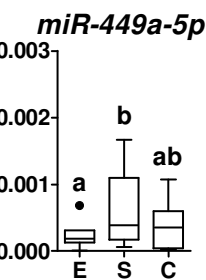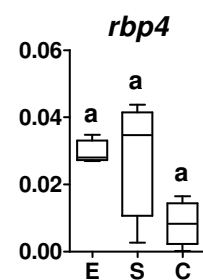

Supplement: Additional file 5 — Comparative expression profile of RNAs related to sex steroid signalling and mucociliary epithelium (MCE) differentiation in whole embryos, isolated tadpole skin or ectodermal explants. [file 1742-9994-11-9-S5.pdf]

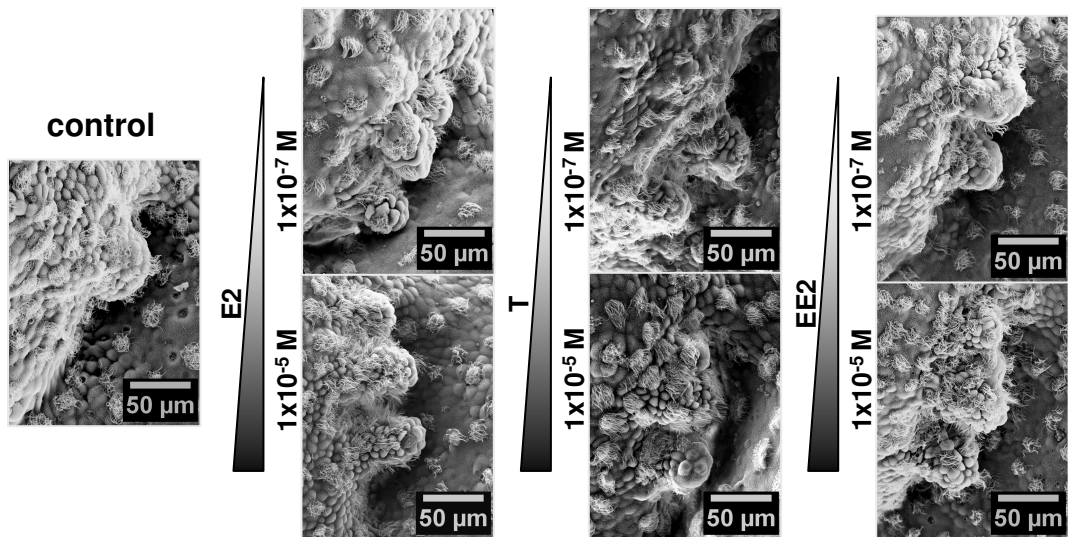

Supplement: Additional file 6 — Effects of estradiol (E2), testosterone (T) and ethynyl-E2 (EE2) on gill branches anatomy in Xenopus embryos. [file 1742-9994-11-9-S6.pdf]

## Slide 1
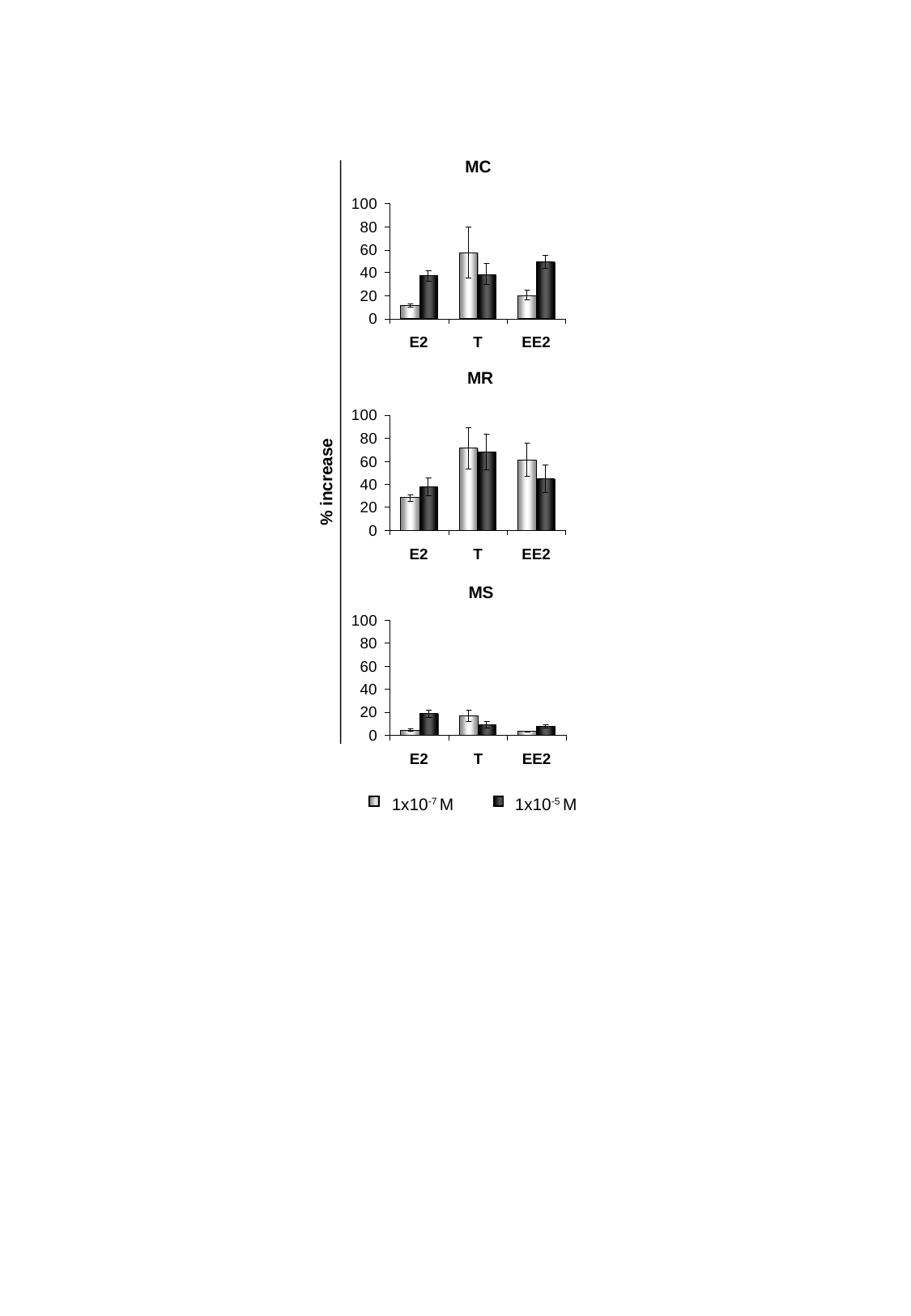

MC
MR
% increase
MS
1x10-7 M
1x10-5 M

Supplement: Additional file 8 — Estradiol (E2), testosterone (T) and ethynyl-E2 (EE2) affect the cellular composition of the Xenopus embryonic skin. [file 1742-9994-11-9-S8.ppt]

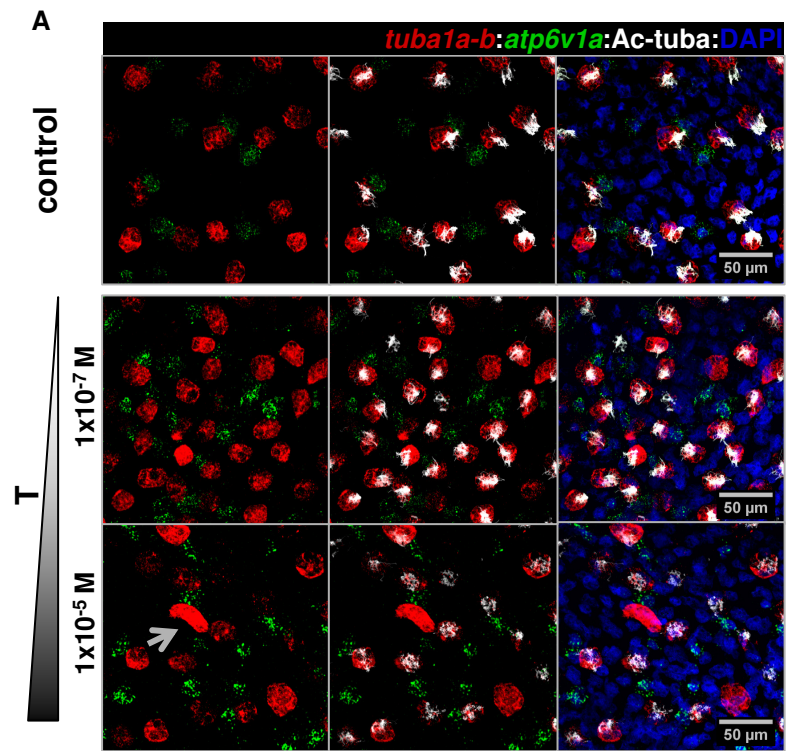

**E2**

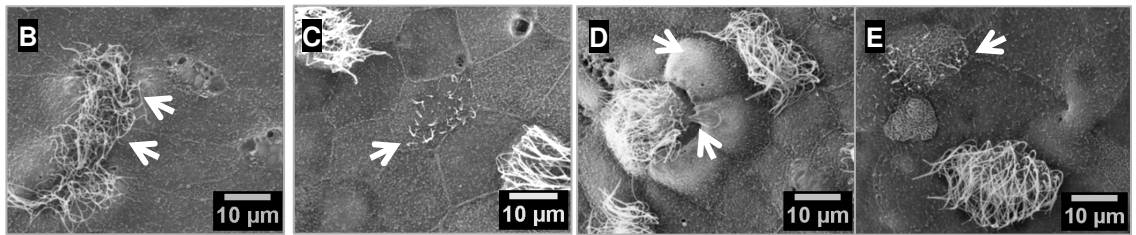

**EE2**

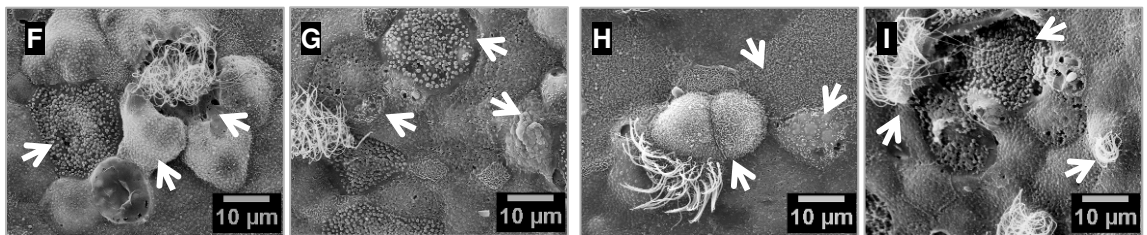

Supplement: Additional file 9 — Effects of sex steroids on the overall arrangement of epidermal MCE in Xenopus embryos. [file 1742-9994-11-9-S9.pdf]

**E2**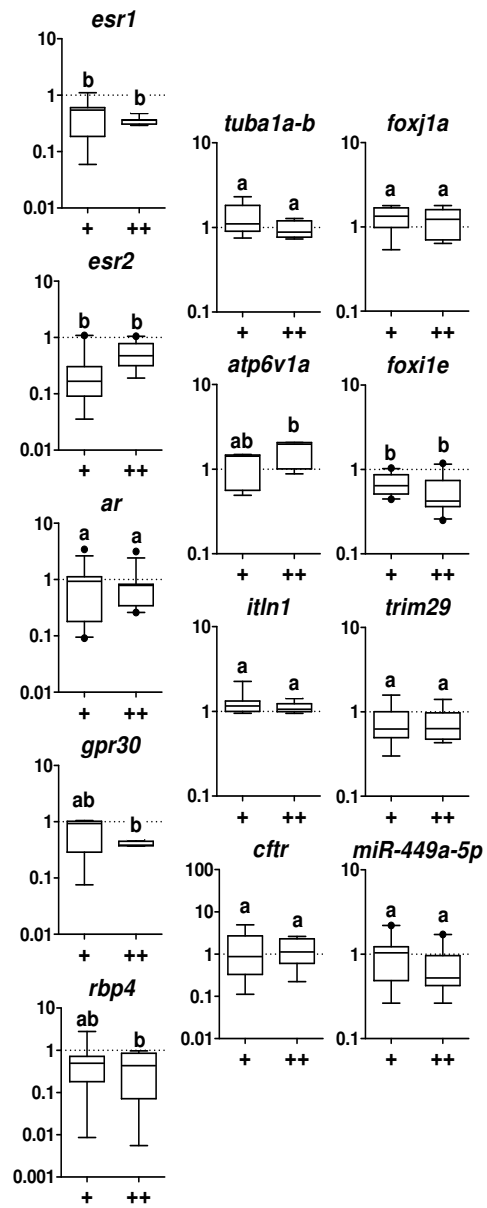**T**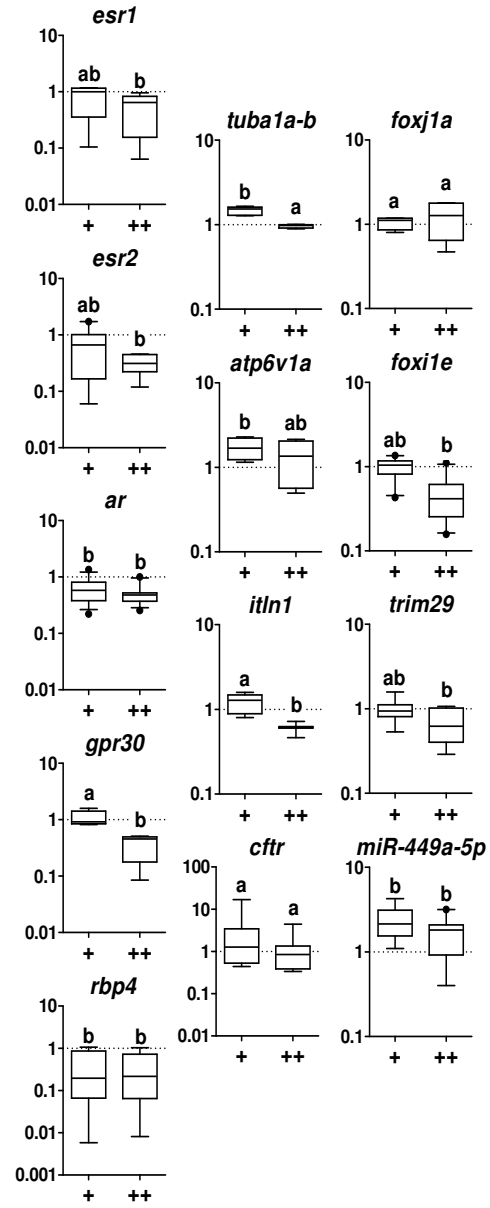**EE2**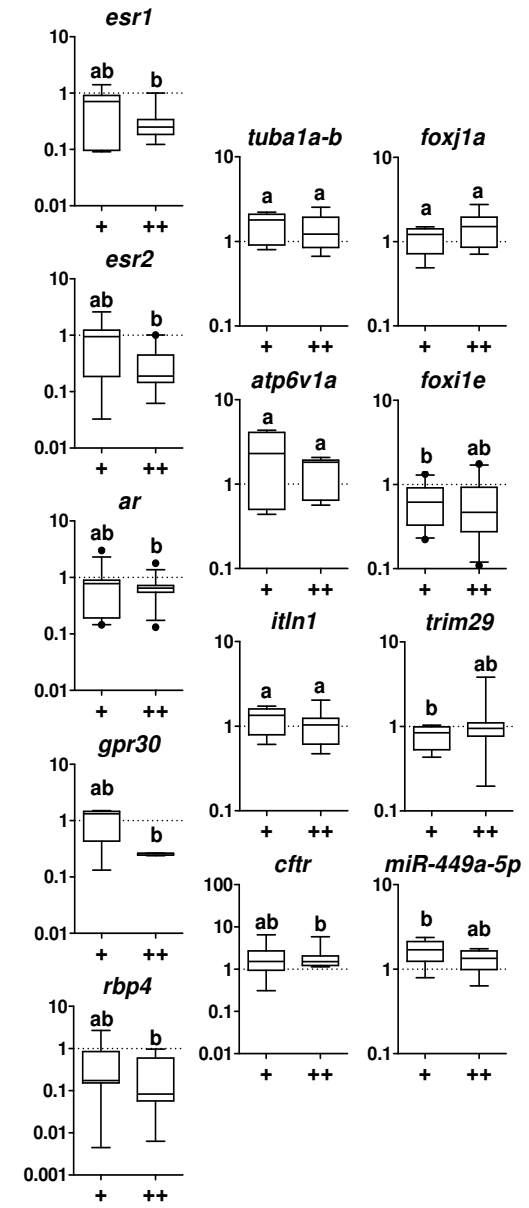

a = control group

Supplement: Additional file 10 — Effects of sex steroids on MCE marker gene expression. [file 1742-9994-11-9-S10.pdf]
