# Supplementary material for: Xenopus embryonic epidermis as a mucociliary cellular ecosystem to assess the effect of sex hormones in a non-reproductive context
Source: Front Zool. 2014 Feb 6;11:9. doi: 10.1186/1742-9994-11-9 (PMC4015847; doi:10.1186/1742-9994-11-9)
Supplement: Additional file 7 — Effects of estradiol (E2), testosterone (T) and ethynyl-E2 (EE2) on the abundance of each cell population of the MCE in Xenopus laevis embryonic skin at st 40 (SEM analysis). [file 1742-9994-11-9-S7.pdf]

Additional file 7. Effects of estradiol (E2), testosterone (T) and ethynyl-E2 (EE2) on the abundance of each cell population of the MCE in *Xenopus laevis* embryonic skin at st 40 (SEM analysis)

	**Control**		**E2**				**T**				**EE2**			
molarity			$1x10^{-7}$		$1x10^{-5}$		$1x10^{-7}$		$1x10^{-5}$		$1x10^{-7}$		$1x10^{-5}$	
**Cell type**	mean	sd	mean	sd	Mean	sd	mean	sd	Mean	sd	mean	sd	mean	sd
MC	23	4	35 [b]	4	41 [b]	10	31 [b]	5	33 [b]	7	33 [b]	8	38 [b]	8
vMR	34	9	38	17	33	12	35	10	36	10	44 [b]	11	35	10
rMR	31	13	46	17	48 [b]	19	53 [b]	13	63 [b]	13	52 [b]	13	54 [b]	14
MS	230	42	232	36	250 [b]	53	244	42	243	39	282 [b]	51	251 [b]	27
**Total**	**318**	**45**	**350**	**69**	**371 [b]**	**84**	**363 [b]**	**52**	**374 [b]**	**49**	**410 [b]**	**68**	**378 [b]**	**44**

[b] The amount of cells was significantly different than in the control group ($p < 0.05$), MC = multiciliated cells, vMR or rMR = vesicle or ridged mitochondrion-rich cells; MS = mucus-secreting cells
