# Supplementary material for: Xenopus embryonic epidermis as a mucociliary cellular ecosystem to assess the effect of sex hormones in a non-reproductive context
Source: Front Zool. 2014 Feb 6;11:9. doi: 10.1186/1742-9994-11-9 (PMC4015847; doi:10.1186/1742-9994-11-9)
Supplement: Additional file 11 — Classification of “non-typical” cellular phenotypes caused by sex steroids in the epidermal mucociliary epithelium of Xenopus. [file 1742-9994-11-9-S11.pdf]

Additional file 11. Classification of "non-typical" cellular phenotypes caused by sex steroids in the epidermal mucociliary epithelium of *Xenopus*.

Cell type	Morphology (SEM)	Molecular markers (*ISH*, IHC)
MC-MS	Cells with cilia and secreting material;<br>Plane polygonal cells with small apical cilia	*tuba1a-b*(+) and *itln1*(+);<br>*tuba1a-b*(+) and Itln1(+);<br>ac-Tuba (+) and *itln1*(+);<br>*tuba1a-b*(-):*atp6v1a*(-) and ac-Tuba(+)
MC-MR	Cells showing ridged surface or apical vesicles and small cilia	*atp6v1a*(+) and *tuba1a-b*(+)
MS-like	Swollen or plane polygonal cells with secreting material on the surface	-
MC-like	-	*tuba1a-b*(+) cells with at least one of the following features: oversized, adjacent between them, ac-Tuba (-) or intracellular ac-Tuba
pMC	-	*tuba1a-b*(+) and pH3(+)
pMR	-	*atp6v1a*(+) and pH3(+)

SEM = scanning electron microscopy, *ISH = in situ* hybridization for marker genes, IHC = immuno-histochemistry for marker proteins, MC = multiciliated, MR = mitochondrion-rich, MS = mucus secreting
