# Supplementary material for: Xenopus embryonic epidermis as a mucociliary cellular ecosystem to assess the effect of sex hormones in a non-reproductive context
Source: Front Zool. 2014 Feb 6;11:9. doi: 10.1186/1742-9994-11-9 (PMC4015847; doi:10.1186/1742-9994-11-9)
Supplement: Additional file 12 — List of Xenopus laevis specific primers used for RT-qPCR analysis. [file 1742-9994-11-9-S12.pdf]

Additional file 12. List of *Xenopus laevis* specific primers used for RT-qPCR analysis

Symbol	**Primers sequences (5' → 3')**<br>Forward (F) / Reverse (R)	Accession number
ar	F: agccgattgcccgtgagctt<br>R: ggctcacccccattgggcatc	NM_001090884.1
atp6v1a	F: tatgtgttggagaccctgtgc<br>R: tggtgtcgctgatgtctttca	NM_001096102.1
cftr	F: aggcctgtcagctggaagagg<br>R: atccttgctcgctggcctcca	X65256.1
esr1	F: tgtgaaaagcatgaagctgagccca<br>R: tggaagcttcactgagtggtttggt	NM_001089615.2
esr2	F: gcgaaccaaagtccattgta<br>R: ggagttgtccacagcaatc	NM_001130954.1<br>[74]
foxj1a	F: cgacatgcagatcccacctggc<br>R: tgcctggctcgtctttttcccg	NM_001090177.1
foxi1e	F: catggagccccagataaaag<br>R: ttgggtccaaggtccaataa	NM_001086898.1<br>[61]
grp30	F: ggtacattgcactggcgaaggtg<br>R: atgcatgtcccctgtgtgctgc	NM_001114253.1
itln1	F: agctgttaacactgagagggc<br>R: attgtctgggacttgcctctg	NM_001089101.1
odc1	F: gccattgtgaagactctctccattc<br>R:ttcgggtgattccttgccac	NM_001086698.1
rbp4	F: atgaccgcaacagctaaagggag<br>R: aatcagtgtccacgacccaatgg	NM_001087726
**RNU2**	Custom LNA primers (Exiqon)<br>for: cuccaggaccggugcacuu	NR_033287.1
trim29	F: tggaaatcagctaagccgaccct<br>R tgccctgctgccataatctgtca	NM_001086106.1
tuba1a-b	F: gtgacccccgtcatggtaaat<br>R cgcttggtttttactgtggca	NM_001086587.1
**xtr-miR-449a-5p**	Custom LNA primers (Exiqon)<br>for: aggcaguguaauguuagcuggu	MIMAT0003704
